# Comparison of Bone Resorption Rates after Intraoral Block Bone and Guided Bone Regeneration Augmentation for the Reconstruction of Horizontally Deficient Maxillary Alveolar Ridges

**DOI:** 10.1155/2016/4987437

**Published:** 2016-10-26

**Authors:** B. Alper Gultekin, Elcin Bedeloglu, T. Emre Kose, Eitan Mijiritsky

**Affiliations:** ^1^Department of Oral Implantology, Faculty of Dentistry, Istanbul University, Istanbul, Turkey; ^2^Oral and Maxillofacial Surgery, Faculty of Dentistry, Aydın University, Istanbul, Turkey; ^3^Department of Oral and Maxillofacial Radiology, Faculty of Dentistry, Istanbul University, Istanbul, Turkey; ^4^Department of Oral Rehabilitation, The Maurice and Gabriela Goldschleger School of Dental Medicine, Tel-Aviv University, Tel Aviv-Yafo, Israel

## Abstract

*Purpose*. Bone atrophy after tooth loss may leave insufficient bone for implant placement. We compared volumetric changes after autogenous ramus block bone grafting (RBG) or guided bone regeneration (GBR) in horizontally deficient maxilla before implant placement.* Materials and Methods*. In this retrospective study, volumetric changes at RBG or GBR graft sites were evaluated using cone-beam computed tomography. The primary outcome variable was the volumetric resorption rate. Secondary outcomes were bone gain, graft success, and implant insertion torque.* Results*. Twenty-four patients (28 grafted sites) were included (GBR, 15; RBG, 13). One patient (RBG) suffered mucosal dehiscence at the recipient site 6 weeks after surgery, which healed spontaneously. Mean volume reduction in the GBR and RBG groups was 12.48 ± 2.67% and 7.20 ± 1.40%, respectively. GBR resulted in significantly more bone resorption than RBG (*P* < 0.001). Mean horizontal bone gain and width after healing were significantly greater in the GBR than in the RBG group (*P* = 0.002 and 0.005, resp.). Implant torque was similar between groups (*P* > 0.05).* Conclusions*. Both RBG and GBR hard-tissue augmentation techniques provide adequate bone graft volume and stability for implant insertion. However, GBR causes greater resorption at maxillary augmented sites than RBG, which clinicians should consider during treatment planning.

## 1. Introduction

Adequate hard tissue around a dental implant is crucial for the long term success of the implant placement. However, unfavorable conditions, due to oral infections, bone atrophy after dental extractions, and long term edentulism, may result in insufficient available bone, making implant placement impossible. A variety of surgical techniques, such as onlay grafts, ridge splitting, distraction osteogenesis, and guided bone regeneration (GBR), have been recommended for the rehabilitation of resorbed alveolar ridges to ensure that implants are placed under optimum conditions [1]. Onlay bone graft applications and GBR have become some of the most common treatment modalities for overcoming hard-tissue defects in preprosthetic surgery [1].

Autogenous bone blocks are still considered the gold standard for the reconstruction of deficient alveolar ridges, because of their osteogenic potential [2]. The use of intraoral autogenous bone blocks has been reported as a reliable and predictable technique for increasing moderately to severely deficient alveolar ridges [3].

Guided bone regeneration is another method for augmenting bone volume and uses barrier membranes containing autogenous bone and/or bone substitutes [1]. The application of resorbable membranes has many advantages, such as easy manipulation, an undemanding flap design, and a reduced risk of membrane exposure, in comparison to nonresorbable membranes [1, 4]. Therefore, in recent years, the use of resorbable collagen membranes for GBR has increased markedly, particularly for horizontal augmentation [1].

Augmented bone stability is considered to be an important factor for the success of the procedure, especially in two-stage regeneration procedures. Bone remodeling has a major influence on long-term clinical outcomes, and graft stability is desirable for integrating dental implants so as to ensure a good outcome [1]. Deproteinized bovine bone (DBB) is an osteoconductive bone substitute that can withstand resorption during healing and can provide a good scaffold for natural bone growth [4]. DBB can be used with autogenous bone and its slow resorption properties could be an advantage in that it helps to maintain the volumetric stability of augmented bone [4].

Little is known about the volumetric extent of resorption of intraoral block bone grafts and GBR augmentation prior to implant placement. Treatment planning could be facilitated if the resorption rate of the grafted bone volume is known, as clinicians can then choose the optimum treatment modality for patients and may not need to perform repeat surgeries to increase bone volume, which has a marked impact on patient morbidity.

The primary aim of the present study was to evaluate the volumetric changes in patients who underwent autogenous ramus block bone grafting (RBG) or GBR in horizontally atrophic maxillae, based on three-dimensional (3D) analysis of cone-beam computed tomography (CBCT) images. More specifically, this study aimed to compare the resorption rates of horizontally augmented alveolar bone between RBG and GBR techniques and to estimate the bone gain achieved before implant placement. The null hypothesis was that there would be no difference between the two interventions in terms of the rate of volume reduction of the grafted bone.

## 2. Materials and Methods

### 2.1. Study Design and Sample Selection

This retrospective study included patients with deficient alveolar ridges who underwent intraoral onlay block bone grafting, using the ramus of the mandible, or GBR, between January 2013 and January 2014, at the Department of Oral Implantology Istanbul University Faculty of Dentistry or the Department of Oral and Maxillofacial Surgery, Aydın University Faculty of Dentistry, Istanbul, Turkey. Subjects were derived from a population of patients with moderate to severe bone resorption and required implant placement in the maxillary alveolar ridge. Sample selection was performed by retrospective chart review.

Inclusion criteria for this study were as follows: the presence of a deficient maxillary ridge requiring two-stage horizontal bone augmentation for dental implant placement; the presence of a residual alveolar ridge with residual bone width < 5 mm and adequate bone height; bone volume at the ramus donor site that allowed harvesting of a block graft; availability of CBCT data acquired before, 3 weeks after surgery, and at last follow-up (healing periods for RBG and GBR were 4 months and 6-7 months, resp.). The exclusion criteria were as follows: lack of CBCT data; previous surgery at the recipient site; systemic diseases that might unfavorably influence soft and/or hard-tissue healing; chronic periodontitis in the remaining teeth; bone defects due to tumor resection; pathologic lesions prior to operation; a history of radiotherapy in the head and neck region; and smoking.

The decision to use GBR or RBG as treatment choice was based on patient-specific anatomical handicaps; for instance, if during treatment planning based on CBCT sufficient autogenous bone particles could be acquired from near the recipient site, GBR treatment was chosen; however, if not, RBG treatment was chosen.

The study protocol followed the Declaration of Helsinki and was approved by the ethical committee of the Aydın University, Turkey (approval protocol number: 480.2/116). Written informed consent was obtained from all patients.

### 2.2. Surgical Methods

All patients were treated with a two-stage approach by either of two surgeons (GBR group: B. Alper Gultekin; RBG group: Elcin Bedeloglu). All surgical procedures were performed under local anesthesia. Prior to surgery, all patients were instructed to rinse their mouths with 0.2% chlorhexidine mouthwash (Chlorhex, Drogsan Pharma, Istanbul, Turkey) for 1 min.

**Figure 1:**
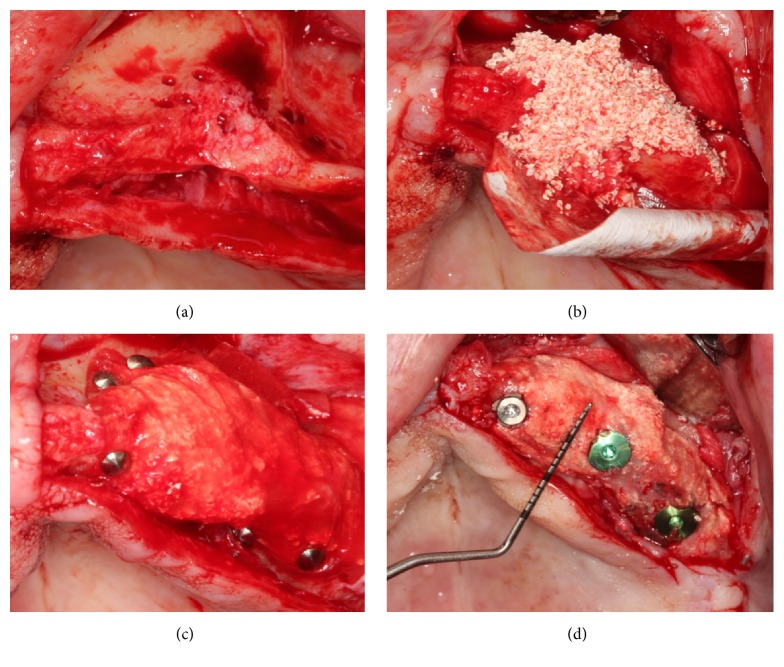
Resorbable collagen membrane and composite graft (autogenous particle bone and deproteinized bovine bone) were applied for horizontal augmentation (a–c); implants were placed after healing (d).

For the GBR group, crestal and vertical incisions were made along the residual alveolar ridge. A mucoperiosteal flap was gently elevated to allow complete visualization of the horizontal defect and the surrounding bone. The native bone was perforated by drilling under saline irrigation, to ensure vascularization between the graft and the recipient site. The recipient bone was curetted to remove any soft tissue that may impede bone healing. Autogenous bone particles were harvested from near the recipient site using a bone scraper (Safe scraper, META, Reggio Emilia, Italy) and mixed with DBB (particle size, 0.25–1.0 mm; Bio-Oss, Geistlich Pharma AG, Wolhusen, Switzerland) in a ratio of approximately 1 : 1 to form the composite graft. Resorbable collagen membrane (Bio-Gide, Geistlich Pharma AG, Wolhusen, Switzerland, or Mem-Lok, Collagen Matrix, Franklin Lanes, NJ, USA) was trimmed according to the contours of the grafting site and then applied for horizontal augmentation. After grafting, the resorbable membrane was immobilized with tacks (Pinfix, Sedenta, Istanbul, Turkey) into the palatinal and buccal sites. Flaps were repositioned with interrupted nonresorbable mattress sutures, with periosteal-releasing incisions (Figure 1).

**Figure 2:**
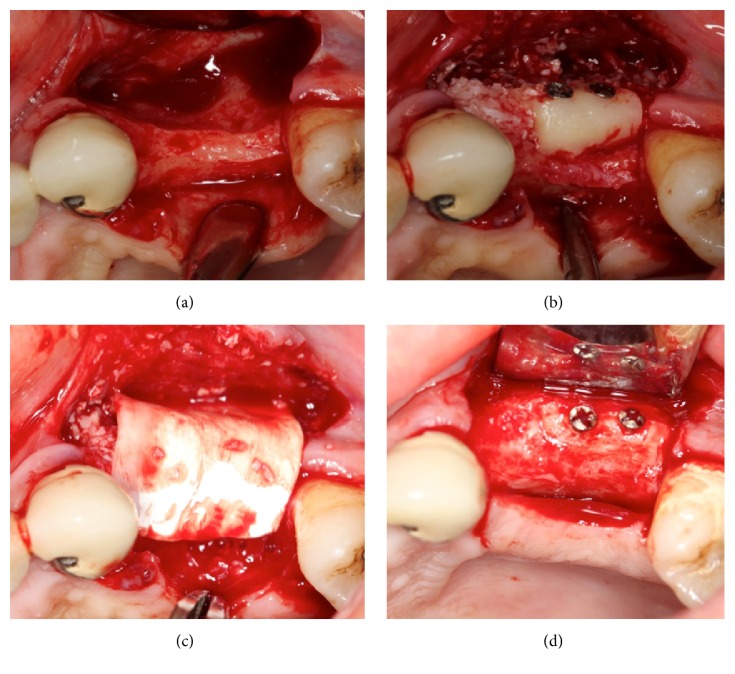
The block bone graft was fixed to the residual ridge with screws and a particulate deproteinized bovine bone graft was used to fill the voids around the block bone and the recipient site (a, b); a resorbable collagen membrane was used to cover the grafted site (c); grafted site after 4 months of healing (d).

For the RBG group, crestal and vertical incisions were made along the residual alveolar ridge at the recipient site. The mucoperiosteal flap was gently elevated to allow complete visualization of the horizontal defect and the surrounding bone. The native bone was perforated by drilling under saline irrigation, to ensure vascularization between the graft and recipient site. To harvest the bone block, infiltration anesthesia was also administered to the left or right donor site. In the ramus zone, midcrestal incision was performed. After reflection of the full-thickness flap and exposure of the donor site, a mandibular block bone was harvested by splitting the outer cortical plate according to the required size to produce a bone block from the retromolar area. In all patients, piezoelectric surgery (Piezon Master, EMS, Basel, Switzerland) or rotary instruments were used, under copious irrigation, to harvest the bone block. A surgical chisel and hammer were used to mobilize the block graft. The block bone graft was recontoured, using a diamond bur, to ensure that it was optimally adapted to the recipient site as an onlay. It was then fixed to the residual ridge, using one or two screws, to inhibit micromovement during healing. Graft corners between the graft and native bone were smoothed to avoid undesirable exposure because of pressure during healing. A particulate deproteinized bovine bone graft (Bio-Oss) was used to fill the voids around the block bone and recipient site. A resorbable collagen membrane (Bio-Gide) was used for covering the graft particles and block bone without tacks (Figure 2). A periosteal-releasing incision was made to allow passive primary closure of the flap. Wound adaptation was achieved with horizontal mattress and interrupted 4–0 nonabsorbable monofilament sutures (Seralon, Serag-Wiesner, Naila, Germany).

All patients were prescribed postsurgical medications, including antibiotics (1000 mg amoxicillin and clavulanic acid, twice daily for a week, starting from the day of surgery), analgesics (600 mg ibuprofen, to be taken per requirement, every 6 h), and 0.2% chlorhexidine mouthwash (twice daily for 2 weeks, starting from the day after surgery). Dexamethasone (4 mg per day) was administered for 3 days to minimize edema. An extraoral cold pressure dressing was applied to minimize postoperative swelling. Oral sutures were removed 3 weeks after surgery. Patients in the RBG and GBR groups were allowed healing periods of 4 and 6-7 months, respectively, before placement of rough-surface dental implants. Patients were prohibited the use of temporary prostheses during the healing period. Patients then received fixed cement-retained porcelain-fused-to-metal crowns and bridges or removable-bar overdenture prosthetic restorations.

### 2.3. Study Variables

The primary predictor and outcome variables were the augmentation technique (RBG or GBR) and the rate of resorption at the augmented site, before implant placement, respectively. Secondary study variables included the success of bone grafting, bone gain, and implant stability.

### 2.4. Clinical Assessment

Patients in both treatment groups were evaluated clinically. Any complications, such as graft or block exposure, infection, immobilization of the block graft, loss of bone particles, and adequate bone volume during implant placement, were evaluated. The clinical success of implant placement at the graft site was evaluated on the basis of implant stability at the second stage surgery. Final insertion torques (< or ≥35 Ncm) of implants during placement at graft sites were recorded using a physiodispenser (W&H ImplantMed, Burmoos, Austria).

### 2.5. Radiographic Assessment

**Figure 3:**
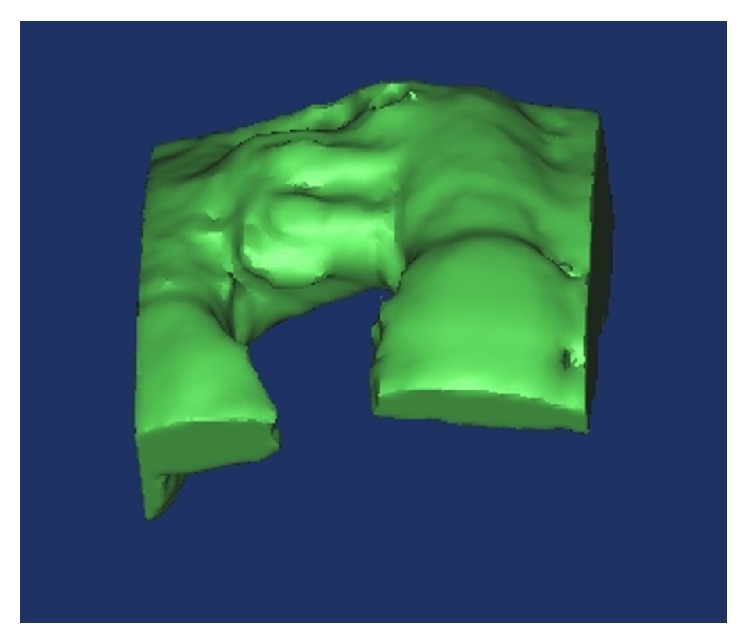
Digital reconstruction was performed by selecting the grafted site, and volumetric changes were analyzed.

Pre- and postsurgical repetitive radiological assessments were performed using CBCT to evaluate volumetric changes at the augmented sites. Images were acquired before surgery, within 3 weeks (V1), and after 4 or 6-7 months after bone grafting (V2), depending on the treatment method. Image analysis was performed using the i-CAT 3D imaging system (Imaging Sciences International Inc., Hatfield, PA, USA), with a field of view of 13 × 8 cm and a voxel size of 0.25. The methodology for digital volumetric calculation has been described earlier [5]. The augmented area was traced as a region of interest. Imaging data of the augmented sites were transferred to a new workstation, where the volumetric changes in bone grafts were analyzed using MIMICS 14.0 software (Materialise Europe, World Headquarters, Leuven, Belgium). Augmented sites were reconstructed in 3D to assess postsurgical volumetric changes at two reference time points (V1 and V2). In order to ensure the reproducibility of volumetric measurements during different time periods, graft sites were selected using anatomical landmarks, fixation tacks, and screws as points of reference (Figure 3). During digital reconstruction of the augmented sites, resorbable membranes, tacks, and native bone, screened at regions of interest in augmented sites, were included in volumetric measurement. Presurgical residual bone width (W0) and augmented bone width (W1) after healing were measured linearly, 2 mm apical to the top of the crest, at a point near the planned implant insertion site, using the i-CAT software. In addition, bone gain was calculated for horizontally augmented sites. A single value of bone gain was anticipated for each graft site. In cases where more than one implant was to be placed at the graft site, the greatest horizontal bone gain was considered for further analysis. All radiographic volumetric and linear measurements were acquired and recorded by the same calibrated independent examiner (T. Emre Kose) under identical conditions, in order to prevent bias and ensure excellent reliability (*R* = 0.964).

### 2.6. Statistical Analysis

Statistical analyses were performed using the Number Cruncher Statistical System 2007 (Kaysville, Utah, USA). Descriptive statistical values were expressed as mean, standard deviation, minimum, maximum, frequency, and percentages. Independent samples *t*-tests were used to test differences in quantitative variables between two independent groups. Yates' continuity correction was used to test differences in qualitative variables between the two independent groups. Pearson's correlation coefficient was used to analyze the correlation among quantitative variables. Linear regression analysis was conducted to analyze the possible risk factors for change in volume (V1-V2). *P* values less than 0.05 were considered statistically significant.

## 3. Results

Of the 26 patients initially enrolled in the study, two were excluded because of the poor quality of imaging data. Eventually, 24 patients with 28 grafted sites (GBR, 15; RBG, 13) were determined to be eligible for inclusion in this study (Table 1). Bilateral augmentation was performed in two patients in each group. In a single patient in the RBG group, mucosal dehiscence was observed at the recipient site at 6 weeks after operation, as a complication. The minor exposed site was removed by using a diamond bur under copious irrigation, and the exposed region disappeared spontaneously in subsequent weeks, without infection. After healing, implants were placed at the graft site, without any complications. Following graft integration, a total of 41 rough-surface dental implants (GBR, 23; RBG, 18) were successfully placed, without encountering any primary stability problems at the reentry stage. Only in one case in the GBR group was contour augmentation (DBB and collagen membrane were used) applied during implant placement, to thicken the buccal bone.

There were no significant differences in patient's age, sex distribution, implant torque values, and presurgical bone width (W0) between the two groups (*P* > 0.05; Table 2). Bone width (W1) and bone gain (W1-W0) after healing in the GBR group were significantly higher than those in the RBG group (*P* = 0.005 and *P* = 0.002, resp.; Table 2).

The mean values of percent volume reduction after healing in the GBR group (12.48 ± 2.67%) were significantly higher than those of the RBG group (7.20 ± 1.40%, *P* < 0.001; Table 3). Although the postaugmentation graft volumes (V1 and V2) of the GBR group were higher than those of the RBG group, no statistically significant differences were found (*P* > 0.05; Table 3).

No significant correlation was found between variables (age, gender, pre- and postsurgical bone width, bone gain, and implant torque) and rate of graft resorption (V1-V2) in the groups (*P* > 0.05, Table 4). No significant correlation was found between the initial postaugmentation bone volume (V1) and the rate of resorption (V1-V2) in GBR and RBG groups separately (*P* > 0.05, Table 4). However, the initial postaugmentation graft volume (V1) and rate of graft resorption (V1-V2) were found to be significantly and positively correlated (*r* = 0.459, *P* = 0.014).

Linear regression analysis was used to identify factors involved in V1-V2 change. The model was found to be statistically significant and variables in the model explained 72.8% of the V1-V2 model variance (*F*: 25.050, *P* < 0.001, *R*_adj_^2^: 0.728, Table 5). When the effect of other variables was held constant, application of RBG rather than GBR resulted in a 6.030 decrease in V1-V2 change (*β* [95% confidence interval, 95% CI]: −6.030% [−7.742%, −4.317%], *P* < 0.001, Table 5). When the effect of other variables was held constant, a unit increase in V1 caused an increase of 0.0086% in the V1-V2 value (*β* [95% CI]: 0.0086% [0.0002%, 0.0015%], *P* = 0.012, Table 5).

## 4. Discussion

A prosthetically driven treatment approach recommends that a deficient edentulous ridge that precludes optimum implant placement requires bone reconstruction [6]. The maxilla is prone to resorption in a centripetal direction; therefore, a deficiency in bone width after tooth loss is very common in the upper jaw. The present study aimed to compare GBR and RBG groups for horizontal deficiency in the maxillary alveolar ridge in terms of the resorption of bone at graft sites and of augmentation treatment success.

Although we observed a significant volumetric reduction in the bone graft in both groups, the extent of resorption during follow-up in the GBR group was greater than that in the RBG group. According to the literature, sites augmented with mandibular block bone have resorption rates between 5% and 28% [2, 6–14]. Cordaro et al. reported resorption of mandibular autogenous block graft sites (22%) in all of their patients at 4 months after maxillary augmentation [11]. In some of their cases, they used DBB and collagen membrane to reduce the resorption rate. Linear measurements were performed using a millimeter-graduated caliper. In another study, Hernández-Alfaro et al. found a 5% resorption rate after total reconstruction of the atrophic maxilla by using intraoral bone blocks and biomaterials [12]. In their study, 3D analysis was performed to measure the changes at the grafted site by means of CBCT scans. Pistilli et al. have reported a 25% bone resorption rate from the initial volume of autogenous onlay blocks [13]. In another study, Lumetti et al. found a 28% resorption rate after ramus or symphysis autologous block bone grafting for augmenting horizontal ridges [14]. In the present study, we generally found less bone resorption in the RBG group than in previous related intraoral block grafting studies [11, 13, 14]. Several factors may influence resorption rates after block bone grafts, such as the type of reconstruction, technique, the cortical bone amount and density at the donor site, biomaterial usage, healing time, and most importantly the measurement method [1, 2, 8–10]. In most previous studies, measurements were made linearly, which induces a high risk of bias.

Mandibular bone blocks are more resistant to resorption due to the vast amount of cortical bone (intramembranous bone graft); however, this advantage may hold a risk in terms of integration of the block and natural bone, due to the limited revascularization and poor regeneration potential of the block [1, 6]. Lozano et al. observed that the revascularization process of a block graft increases with time [15]. In the present study, we waited 4 months to enhance vascularization and integration of graft, and we did not observe any complications related to block disintegration during implant placement. All blocks were used in deficient maxillae; blood supply to the maxilla may be better than that to the mandible, which may be another reason for the good integration during healing [13]. Block bone coverage of the recipient site with bone substitutes with low turnover rates, such as DBB and resorbable collagen membranes, may reduce the rate of bone resorption after block bone grafting [4]. Maiorana et al. found that DBB coverage of onlay block grafts reduced resorption by almost 50% in comparison to that in the absence of coverage [16, 17]. Bone substitutes may also contribute to the creation of a smooth connection between bone block and natural bone and can provide a scaffold for the regeneration of bone at these gaps [4, 16]. Another advantage of using resorbable rather than nonresorbable membranes is the elimination of second stage surgery. Although the barrier function cannot be controlled by the clinician and space maintenance is limited, it is likely that the use of resorbable membrane with tacks in the GBR group and without tacks in RBG group would be suitable for the reconstruction of deficient sites.

Bone gain and survival rates of implants in sites grafted using the GBR treatment approach are well documented; however, the stability of regenerated bone has been assessed in very few studies [18, 19]. In the present study, we found more resorption in the GBR group than in the RBG group, but the resorption rate was lower than in other GBR-related bone resorption studies [18, 19]. Mordenfeld et al. found 37% to 46% resorption rates after lateral augmentation with a GBR approach, using two different compositions of graft materials [18]. In their study, composite grafts (DBB and autogenous bone) were covered with collagen membranes, without any fixation. Although they used CBCT scans for measurement, they calculated the changes in graft volume as the product of slice thicknesses of the region of interest and the sum of volumes, rather than obtaining measurements as a single unit [18]. In another study, Sterio et al. observed the resorption or displacement of 50% of horizontal graft material after 6 months of healing [19]. The authors used cancellous allografts and collagen membranes without tacks in order to increase bone width and evaluated the changes in bone dimension by CBCT and 2D measurements using calipers. Proussaefs and Lozada observed a 15.11% resorption rate at 6 months after bone grafting using a composite (DBB and autogenous bone particles) and nonresorbable membrane [20], based on linear measurements made on laboratory casts derived from intraoral impressions.

In the present study, a 12.5% rate of resorption was found for the GBR group after healing. One of the reasons for the reduced resorption observed in the GBR group may be that tacks were used to squeeze the particulate composite graft under the membrane to mimic a block graft to ensure space maintenance and resist the pressure that may be induced by the flap, cheek, or other forces during healing [21]. In the RBG group, space maintenance is achieved by the block itself, and therefore resorbable membrane can be used without tacks and prevent cells, such as epithelial cells, and connective tissue from impeding bone regeneration. Another reason for the reduced resorption of GBR is that it involves a composite of a low turnover graft material and autogenous particulate bone. Autogenous bone particles may accelerate integration with graft particles and decrease the volume reduction of the grafted bone. During healing, vascularization may initiate from perforated residual bone. During drilling and implant placement, the bone appeared to be in a good state, and composite graft particles had become well integrated. It seems that 6-7 months of healing may be sufficient for the formation of a rigid grafted bone that can facilitate implant stability in horizontally deficient ridges. However, one of the major drawbacks of GBR compared to RBG is the necessity for a longer healing period. RBG may be a better option requiring a substantially shorter treatment time than GBR, when time is critical for clinicians and patients.

Dasmah et al. compared graft resorption rates after using autogenous iliac particulates and a block bone treatment approach in the reconstruction of atrophic maxilla [22]. Although they found no statistical difference between the two groups, a marked resorption rate (80%) was observed in both groups. In the present study, we found lower resorption rates than those reported by Dasmah et al. [22]. Usage of intraoral sources, such as the ramus or symphysis for block grafting, and biomaterials, such as autogenous particulate grafts in the GBR approach, seems to eliminate unpredictable resorption. Another possible reason for the lower rate of resorption in both groups in the present study than in previous studies involving GBR and RBG is that we did not use removable provisional restorations during the healing stage. It is well known that any soft tissue support prosthesis may increase the resorption of both the residual and grafted bone [23]. In light of the promising results in terms of the GBR resorption rates observed in the present study, we speculate that a collagen membrane, composite graft, and tacks can offer an alternative to nonresorbable membranes. The latter membranes have many disadvantages, such as a high risk of wound infection, requirement for second stage surgery, and a long learning curve in terms of reconstruction of horizontal defects, before implants can be placed.

Both groups in the present study exhibited adequate horizontal bone gain for implant placement after the healing period. There is a great discrepancy in the literature about the extent of horizontal bone gain after bone augmentation with RBG and GBR. Previous studies involving two-stage approaches have reported a mean horizontal bone gain ranging from 4 to 6 mm after RBG [3, 4, 7, 10, 24, 25], while the mean horizontal bone gain in GBR approaches has been reported to range from 1.37 to 6 mm [18, 19, 21, 26, 27]. Our results for both groups are in agreement with those of previous studies. In the present study, the GBR group demonstrated significantly greater bone gain for horizontal augmentation than did the RBG group after healing. In the RBG group, the maximum cutting depth of the bone block is limited by the anatomical restrictions of the lower jaw; therefore, horizontal bone gain is of necessity directly proportional to the thickness of the harvested bone block. In the GBR group, horizontal bone gain can be increased with the amount of composite graft used. However, clinicians should consider the differences in the extent of graft resorption when choosing between these two different treatment approaches.

The present study reported predictable and reliable results for horizontal reconstruction of the maxilla and achieved 100% implant stability at these augmented sites. This result is in accordance with many studies [1, 6, 11]. In the present study, graft sites reconstructed by both treatment approaches had exhibited deficiency in the horizontal dimension. Augmentation of the bone resulted in some part of the implant body being in contact with matured bone, which would increase the primary stability during placement and consequently reduce the risk of implant stability failures. Another reason for enhancing the primary stability of the implants is the placement of implants in a well-revascularized and healed, rigid, grafted area using a two-stage approach. Healed grafted sites may thus have enhanced potential for implant stability [1, 11, 15, 16].

We did not observe any complications, such as infection, temporary or permanent sensory disturbance, or membrane exposure in our patients. Only in the RBG group was the minor dehiscence of the mucosa at the recipient site observed in one case, but this was managed after removing the exposed area. Complications following block bone harvesting at the ramus, as compared to other intraoral donor sites, such as the symphysis, are less common [28]. In the present study, preoperative treatment planning was meticulously performed based on 3D images obtained by CBCT in both groups, and all anatomical restrictions, such as the mandibular alveolar nerve, and the thickness of the buccal cortical bone in retromolar areas were evaluated before harvesting the block bone in RBG group. It may not be possible to make such an extensive evaluation using two-dimensional (2D) radiographs. It can be speculated that both treatment approaches are safe and reliable when using 3D radiographic preoperative evaluation.

In the present study, treatment outcomes were evaluated in 3D using CBCT, rather than making linear measurements by caliper, periodontal probe, or 2D radiographs, such as panoramic radiography. 2D techniques do not provide adequate and reliable measurements for the evaluation of volumetric changes in alveolar crest grafts over time. Additionally, these techniques do not have the ability to measure 3D changes precisely [6, 21]. It can be speculated that CBCT is a reliable and predictable 3D radiographic technique for acquiring high-quality volumetric measurements after ridge augmentation.

One of the limitations of the study is that graft resorption was evaluated during the healing stage only. Nevertheless, bone resorption is expected to be greater before implant placement and loading and to slow significantly thereafter [6, 23]. Therefore, evaluation of resorption is more important before implant placement. Another limitation is the lack of histological analysis in both groups after healing. Nevertheless, the present study provides valuable insights into the volumetric resorption after two intraoral surgical techniques before implant placement.

## 5. Conclusion

It may be concluded that the use of both RBG and GBR for hard-tissue augmentation provides an adequate volume of bone and stability for implant insertion. However, GBR results in greater resorption at maxillary augmented sites than RBG. Therefore, clinicians should consider the differences in the extent of graft resorption when planning treatment.

## Figures and Tables

**Table 1:** Descriptive summary of the study sample.

Study variable	Descriptive statistics
*Sample size*	
Patients, *n*	24
Sites, *n*	28
*Demographic variables*	
Gender	
M/F, *n* (%)	11 (39.3)/17 (60.7)
Age (years), mean ± sd (min–max)	48.82 ± 10.17 (28–67)
*Health status variables*	
ASA classification	
I	24 (100%)
Groups: numbers and sites, *n* (%)	
GBR horizontal	15 (53.6)
RBG horizontal	13 (46.4)
Implant torque, *n* (%)	
Up	15 (53.6)
Down	13 (46.4)
Edentulism, *n* (%)	
Total	8 (28.5)
Partial	20 (71.5)
Prosthesis design, *n* (%)	
Fixed	20 (71.4)
Removable	8 (28.6)

ASA, American Society of Anesthesiology; GBR, guided bone regeneration; RBG, ramus block bone graft.

**Table 2:** Study variables versus predictor variable (augmentation technique).

	GBR (*n* = 15)	RBG (*n* = 13)	*P*
	Mean ± SD	Mean ± SD	
Patient number	13	11	
Graft sites	15	13	
Age, years	48.73 ± 10.96	48.92 ± 9.61	^a^0.962
Gender, F/M, *n*			
Male	5 (33.3)	6 (46.2)	^b^0.761
Female	10 (66.7)	7 (53.8)	
Implant torque, sites			
Up	6 (40.0)	9 (69.2)	^b^0.243
Down	9 (60.0)	4 (30.8)	
W0, mm	3.51 ± 0.70	3.42 ± 0.60	^a^0.720
W1, mm	8.93 ± 0.93	7.96 ± 0.71	^a^0.005^*∗∗*^
W1-W0, mm	5.42 ± 0.76	4.54 ± 0.59	^a^0.002^*∗∗*^

^a^Independent samples *t*-test; ^b^Yates' continuity correction; ^*∗∗*^ *P* < 0.01.

GBR, guided bone regeneration; RBG, ramus block bone graft; W0, presurgical bone width; W1, bone width after healing; W1-W0, bone gain after healing.

**Table 3:** Association between predictor (augmentation technique) and primary outcome (resorption) variable.

	GBR (*n* = 15)	RBG (*n* = 13)	*P*
	Mean ± SD	Mean ± SD	
V1, mm^3^	5557.50 ± 1060.73	4959.11 ± 1152.21	^a^0.164
V2, mm^3^	4853.61 ± 885.61	4594.13 ± 1035.67	^a^0.481
V1-V2 (%)	12.48 ± 2.67	7.20 ± 1.40	^a^<0.001^*∗∗*^

^a^Independent samples *t*-test; ^*∗∗*^ *P* < 0.01.

GBR, guided bone regeneration; RBG, ramus block bone graft; V1 and V2, initial postaugmentation and posthealing graft volumes, respectively; V1-V2 (%), resorption rate.

**Table 4:** Study variables versus primary outcome (resorption) variable.

	V1-V2 (%)	
	Mean ± SD	*P*
Gender, F/M, *n*		
Male	10.11 ± 3.25	^a^0.924
Female	9.98 ± 3.64	
Implant torque, sites		
Up	9.28 ± 3.19	^a^0.219
Down	10.87 ± 3.61	

	*r*	*P*

Age, years	0.105	0.597
W0, mm	0.110	0.576
W1, mm	0.252	0.196
W1-W0, mm	0.210	0.283
V1		
GBR	0.387	0.154
RBG	0.541	0.056
Total	0.459	0.014^*∗*^

^a^Independent samples *t*-test; *r*: Pearson's correlation coefficient; ^*∗*^ *P* < 0.05.

V1-V2 (%), resorption rate; W0, presurgical bone width; W1, bone width after healing; W1-W0, bone gain after healing; V1, initial postaugmentation graft volume.

**Table 5:** Linear regression analysis to identify predictors of V1-V2 change.

	*β*	*P*	95% CI for *β*	
			Lower bound	Upper bound
Constant	21.499	<0.001^*∗∗*^	13.606	29.392
Augmentation technique (RBG)	−6.030	<0.001^*∗∗*^	−7.742	−4.317
V1	0.0086	0.012^*∗*^	0.0002	0.0015

^*∗*^ *P* < 0.05; ^*∗∗*^ *P* < 0.01.

RBG, ramus block bone graft; V1, postaugmentation graft volume; CI, confidence interval.
